# Safety of Natural Insecticides: Toxic Effects on Experimental Animals

**DOI:** 10.1155/2018/4308054

**Published:** 2018-10-16

**Authors:** Abdel-Tawab H. Mossa, Samia M. M. Mohafrash, Natarajan Chandrasekaran

**Affiliations:** ^1^Pesticide Chemistry Department, National Research Centre (NRC), 33 El Bohouth Street (Former El Tahrir St.), P.O. Box 12622, Dokki, Giza, Egypt; ^2^Centre for Nanobiotechnology, Vellore Institute of Technology, Vellore, Tamil Nadu 632014, India

## Abstract

Long-term application and extensive use of synthetic insecticides have resulted in accumulating their residues in food, milk, water, and soil and cause adverse health effects to human and ecosystems. Therefore, application of natural insecticides in agriculture and public health sectors has been increased as alternative to synthetic insecticides. The question here is, are all natural insecticides safe. Therefore, the review presented here focuses on the safety of natural insecticides. Natural insecticides contain chemical, mineral, and biological materials and some products are available commercially, e.g., pyrethrum, neem, spinosad, rotenone, abamectin,* Bacillus thuringiensis* (*Bt*), garlic, cinnamon, pepper, and essential oil products. It can induce hepatotoxicity, renal toxicity, hematotoxicity, reproductive toxicity, neurotoxicity, and oxidative stress. It can induce mutagenicity, genotoxicity, and carcinogenicity in mammals. Some natural insecticides and active compounds from essential oils are classified in categories Ib (Highly hazardous) to U (unlikely toxic). Therefore, the selectivity and safety of natural insecticides not absolute and some natural compounds are toxic and induce adverse effects to experimental animals. In concussion, all natural insecticides are not safe and the term “natural” does not mean that compounds are safe. In this respect, the term “natural” is not synonymous with “organic” and not all-natural insecticide products are acceptable in organic farmers.

## 1. Introduction

Pesticides are playing important role in agriculture and public health. They make an important role by increasing the production of food and fiber and improving human health by reducing the rate of vector-borne diseases [1]. In addition to crop damage induced by pests, these pests that cause adverse effects on human health and domestic animals produce the toxic metabolites. In this respect, according to* The Pesticide Manual *about 812 active ingredients “pesticides” were registered until year 2000 [2]. Today's more than 10,400 pesticides are approved worldwide [3]. It has been reported that the consumption of pesticides accounts two million tons every year worldwide [4]. However, the word “pesticides” represented numerous type of pesticides (e.g., insecticides, herbicides, bactericides, nematicides, acaricides, fungicides, molluscicides, and rodenticides); each is active against specific pests (e.g., insect, weed, bacteria, nematode, fungi, snail, and rat).

Insecticides contain two types; the first is synthetic insecticides assigned to groups based on the mode of toxic action, such as groups of organochlorines, organophosphates, carbamates, and pyrethroids insecticides; the second is natural insecticides such as azadirachtin, rotenone, spinosad, and abamectin. The extensive and long-term application of synthetic insecticides has resulted in accumulating their residues in food, milk, water, soil, and other environmental components. It cause adverse health effects to human and ecosystems. Previous studies showed that synthetic insecticides such as malathion, methomyl, chlorpyrifos, pirimiphos-methyl, dimethoate, and *β*-cyfluthrin caused oxidative stress and liver and kidney damage in experimental animals [5–10]. It caused biochemical and hormonal alteration in sprayers of cotton fields [11] and occupational exposure to pesticides accounts 4% of all human cancers [12].

Natural insecticides contain chemical, mineral, and biological materials and some products are available commercially, e.g., pyrethrum, neem, spinosad, rotenone, abamectin,* Bacillus thuringiensis* (*Bt*), garlic, cinnamon, pepper, and essential oil products [13–19]. Practically, the major categories of natural insecticides are botanical, soaps and oils, minerals, and microbial. The selectivity and safety of natural insecticides are not absolute and some natural compounds are toxic; for example, arsenic and nicotine are used historically as natural pesticides. Currently, these natural compounds are not considered as safe and not used as pesticides.

Therefore, the review presented here focuses on the safety of natural insecticides on experimental animals. Scientific databases were used to search for articles in this review.

## 2. Acute Toxicity Hazard Categories of Pesticides

According to the World Health Organization [20], pesticides were classified by hazard into five classes (Ia, Ib, II, III, and U) based on acute oral or dermal LD_50_ mg/kg. body weight (b.wt.) for the rat (Table 1). Class Ia is classified as extremely hazardous which content pesticides with acute oral LD_50_ less than 5 mg/kg. body weight (< 5) and dermal LD_50_ less than 50 mg/kg. body weight (< 50). Pesticides in other categories, Ib (Highly hazardous), II (Moderately hazardous), and III (Slightly hazardous), have acute oral toxicity 5–50, 50–2000, and over 2000 mg/kg. b.wt. and acute dermal toxicity 50–200, 200–2000, and over 2000 mg/kg. b.wt., respectively. In contrast, category U is classified as pesticides which are unlikely to present acute hazard and have acute oral or dermal toxicity 5000 mg/kg. b.wt. or higher.

## 3. Mechanism of Action of Some Common Natural Insecticides

Several authors reported the mechanism of toxic action of different natural insecticides. The correlation between chemical structure and toxicity also was studied. In this part, the mechanisms of action of natural insecticides (pyrethrins, azadirachtin, spinosad, abamectin, mineral oils,* Bacillus thuringiensis, Lecanicillium muscarium,* and phosphine gas) will be discussed.

### 3.1. Pyrethrins (Pyrethrum)

**Figure 1:**
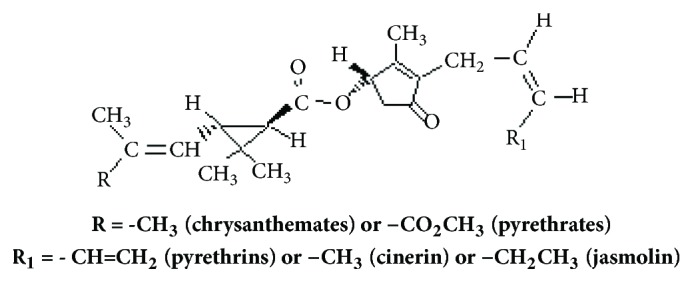
Chemical structure of pyrethrins (pyrethrum).

Pyrethrins (pyrethrum) are a mixture of natural chemical compounds found in the extract of* chrysanthemum* flowers [*Tanacetum *(=* Chrysanthemum = Pyrethrum*)* cinerariaefolium*]. Pyrethrum extract has six different compounds with insecticidal activity (Figure 1). These active compounds in the chrysanthemum flower extract are named pyrethrins [21]. Moreover, pyrethrins are registered as pesticides and more than 2000 commercial products are found worldwide [22]. Pyrethrins like other pyrethroids insecticides are neurotoxicant [23]. It modifies the function of voltage-gated sodium channels [24]. Pyrethrins can cause repetitive nerve impulses by alteration the permeability of excited nerve cells to the sodium ion [23, 25]. It causes other neurobiological effects on gamma amino butyric acid (GABA), noradrenergic, dopaminergic, and cholinergic neurotransmission [26].

According to* The Pesticide Manual* [27], the acute oral toxicity of pyrethrins LD_50_ is 2370 and 1030 mg/kg for male and female rats and 273-796 mg/kg for mice. However, according to the WHO recommended classification of pesticides [20], the active ingredient of pyrethrins insecticide is classified in class II as moderately hazardous.

### 3.2. Azadirachtin

**Figure 2:**
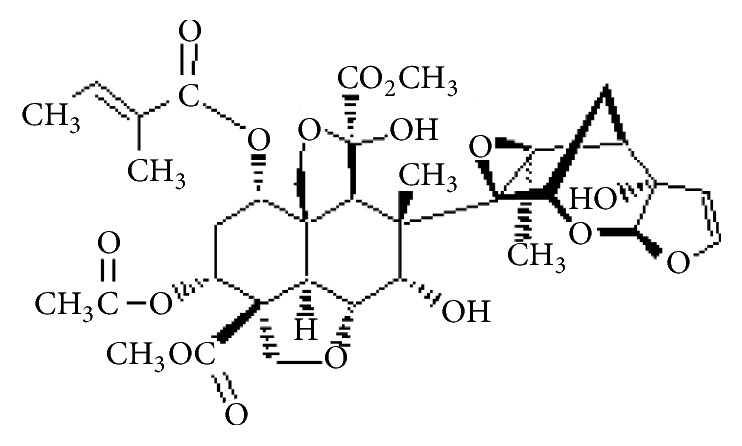
Chemical structure of azadirachtin.

Azadirachtin is the principal natural insecticidal ingredient isolated from neem seed extracts of the neem tree (*Azadirachta indica* A. Juss) [28]. It causes growth disruption, antifeedant, and toxicity to insects [29]. This effect is due to the presence of a complex tetranortriterpenoid limonoid as a major compound (Figure 2). Another variety of limonoids is found in these extracts, e.g., salannin, nimbin, and nimbolide. According to* The Pesticide Manual *[27], the emulsion of neem is prepared from extracts containing azadirachtin (25%) and other limonoids (30-50%) w/w as active compounds. According to Mordue and Blackwell [30], the mechanism of insecticidal action “mode of action” of azadirachtin is due to (i) antifeedancy as result of effect of azadirachtin on deterrent and chemoreceptors (chemosensor); (ii) effect on juvenile and ecdysteroid hormone; (iii) direct effects on most insect tissues. The acute oral toxicity of azadirachtin LD_50_ for rats is more than 5000 (>5000) mg/kg and formulation is classified in class IV according to EPA [27].

### 3.3. Spinosad

**Figure 3:**
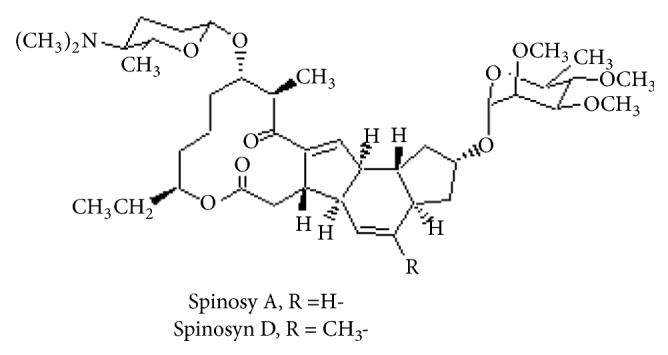
Chemical structure of spinosad.

Spinosad is a natural and active insecticide produced by a soil actinomycete bacterium,* Saccharopolyspora spinosa* [31]. Spinosad contains a number of metabolites that have been named spinosyns (Figure 3). Spinosyns are a mixture of two active compounds spinosyn A and spinosyn D obtained from* S. spinosa* fermentation [32, 33].

Due to the low toxicity and safety to beneficial organisms, spinosad is considered as selective insecticides. Spinosad is a neurotoxin with novel mechanism of action by activation of the nicotinic acetylcholine receptor and gamma amino butyric acid (GABA) receptor [16] but at a different site of action from imidacloprid and nicotine. It is toxic active by ingestion or stomach action and contact. The acute oral toxicity (LD_50_) for male is 3783 and >5000 mg/kg for female rats [27].

### 3.4. Abamectin

**Figure 4:**
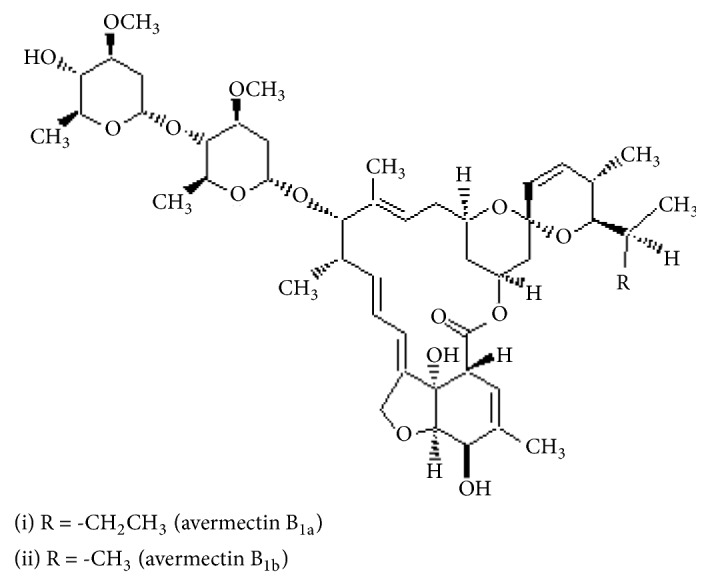
Chemical structure of abamectin.

Abamectin (ABA) is a natural insecticide with stomach action and contact. It has been produced by fermentation of* Streptomyces avermitilis*. The commercial product of abamectin contains two active compounds ≥ 80% avermectin B1a and ≤ 20% avermectin B1b (Figure 4). ABA is a neurotoxin with the specific mechanism of action [34]. It is effected in the nervous system by blocking the ion tropic *α*-amino butyric acid (GABA) [35]. Abamectin has insecticidal, acaricidal, and nematocidal activity. The acute oral LD_50_ of abamectin is 10 mg/kg for rats in sesame oil and 221 mg/kg in water. LD_50_ for mice is 13.6 mg/kg in water [27]. According to WHO [20], it is classified in class II as moderately hazardous.

### 3.5. Mineral Oils

Mineral oils also called petroleum oils, paraffin oils, spray oils, white oils (refined grades), and adjuvant oils. Mineral oils contain mostly saturated and unsaturated aliphatic hydrocarbons. The mineral oils used as insecticides generally distill at temperature above 310°C. These oils are produced by distillation and modification of crude mineral oils. Mineral oils are classified by the proportion distilling at 335°C to light (67-79%), medium (40-49%), and heavy (10-25%). Moreover, adjuvant oils are highly developed self-emulsifying oils, which produce a quick-breaking emulsion, spread rapidly, and support penetration of the active ingredient into the plant and pest.

The mode of toxic action of mineral oils is different from synthetic insecticides, which depend on physical and chemical properties of mineral oils. Moreover, physiological, anatomical, and behavioral characters of insect are playing essential role of mineral oil toxicity. According to Smith [36], the mechanism of toxic action of mineral oils when applied to eggs and used as ovicidal is due to (i) stopping the normal exchange of gases; (ii) stopping hatching due to change the water balance of the egg; (iii) dissolving the outer covering of the egg; (iv) penetrating the egg causing coagulation of the protoplasm; (v) interfering with enzyme or hormone activity. In contrast, when mineral oils are applied as insecticides, they cause toxic action by changes in insect tissue structure as a result to the fumigant effect of volatile compounds [37].

### 3.6. Bacillus thuringiensis (Bt)

Bt is a special bacterium which can produce a number of active chemical compounds that are used commercially as insecticides to control insects in agriculture and public health sectors. The widespread use of* Bt* is mainly due to safety for humans and is environmentally friendly biopesticide worldwide;* Bt* cause mechanism of toxic action by insecticidal activity as stomach action. After application of* Bt* and ingestion by insect, the crystals of endotoxin are solubilized and cause damage in the epithelial cells of the gut. As a result, insects stop feeding and finally starve to death [38, 39].

### 3.7. Lecanicillium muscarium

The fungal pathogen.* L. muscarium* is grown naturally in soils. It is active against a wide-range of insects in different species. It is a good and common pathogen infecting insect hosts worldwide under different geographical and climatic sites.* L. muscarium* commercial product has been in the market since 1980 [40]. It is active against whiteflies on different crops [41]. It was isolated from whiteflies, aphid, and other insect hosts. Previous studies stated that* L. muscarium* are reported as pathogens against different insects worldwide [42–44]. It has been reported that pathogenicity of* Lecanicillium muscarium* is caused by contacting the spores to the insect, germination on insect cuticle, penetration, and inside colonization culminating in host death [45].

### 3.8. Phosphine

Aluminum phosphide and magnesium phosphide were used as a source of phosphine gas for fumigation. The phosphine gas is active against a wide-ranging of insects.

Phosphine has an effect on respiratory, metabolic, and nervous system. It causes nerve poison. The acute oral toxicity (LD_50_) for rats of aluminum phosphide is 8.7 mg/kg and for magnesium, phosphide is 11.2 mg/kg [27]. Due to high toxic effect of aluminum and magnesium phosphide on human, they restricted use of these chemicals.

## 4. Toxicity of Some Essential Oil Compounds to Mammals

Essential oils (EOs) are obtained from different part of plants by hydrodistillation and other methods. Plants in Lamiaceae family were reported as the high botanical sources. EOs are found generally in plants as mixtures of various active components especially monoterpenes (phenols and sesquiterpenes). However, several active compounds were isolated as major compounds from many plants such as 1,8-cineole (rosemary and eucalyptus), eugenol (clove oil), thymol (garden thyme), menthol (mint), asarones (calamus), carvacrol, and linalool (many plant species). Some EOs were used for protection of stored products and some have fumigant and contact insecticidal activities [46–48].

Some of these active compounds have toxic effect to mammals for example; the acute lethal dose (LD_50_) of Thujone is 87.5 mg/kg. b.wt. to mice through subcutaneous route and 500 mg/kg. b.wt of Apiol to dogs through intravenous route. However, Table 2 shows the acute lethal dose of some active compounds [47, 49, 50] and classification according to the WHO acute toxicity hazard categories [20]. Some of these active compounds have toxic effects to experimental animals and have been found in categories II and III.

## 5. Adverse Effects of Natural Insecticides on Experimental Animals

Several scientist studied the adverse toxic effect of natural insecticides on experimental animals. These effects include hepatotoxicity, hematotoxicity, renal toxicity, genotoxicity, and carcinogenicity. The effect on animals at critical period such as pregnancy, lactation, and weanling also was studied. The following is a summary of the studies conducted on some natural insecticides.

### 5.1. Body Weight and Relative Organs Weights

Body weight, weight gains, and organ weights are good criteria for studying the toxicity of xenobiotic in mammal's body and organs. The abnormal changes are used as an indicator of organ dysfunction, detoxification process, and toxicity (Table 3). According to USEPA [51], pyrethrins are slightly toxic to small mammals with an LD_50_ 700 mg/kg. b.wt. It causes a reduction in body weight gain in pups during lactation period and the lowest-observed-adverse-effect level (LOAEL) is 65 mg/kg/day based on decreased body weight. Springfield* et al*. [52] reported that oral administration of pyrethrum at 200 mg/kg for 23 days to male rats increased body and liver weights with decrease in hepatic DNA concentrations. Other studies reported change in body and organ weights of mice [53], rats [54, 55], and dogs [56].

Significant decrease in body weight gain and increase in relative weight of liver and kidney was recorded in mice administrated abamectin at 1/10 LD_50_ (3.802 mg/kg. b.wt.) for 14 days [57]. The authors reported that the decrease in body weight might be due to the decrease in food consumption and or increase degradation of protein and lipids [58, 59]. To study the effect of breastfeeding exposure to the ABA on body and organ weights of mothers and their officering, rat mothers were given ABA orally at doses of 22.10, 11.05, and 2.21 mg/kg. b.wt from postnatal day 1 (PND1) until day 20 (PND20). High dose of ABA (22.10 mg/kg. b.wt.) induced high mortality in male and female suckling rats by 67.5% and 55.0%, respectively [60]. Exposure to the ABA at dose of 22.10 mg/kg. b.wt. induced signs of toxicity and increase in relative liver weight and body weights of mothers and their male and female pups. The authors reported that the decrease in body weight could be due to the neurotoxic action of ABA and their toxic metabolites. This neurotoxic effect leads to increase the degradation of protein and lipids [60–62]. Earlier studies reported decrease and increase in body weight, body weight gain, and relative organ weights of experimental animals exposed to insecticides [63, 64]. Due to its high lipophilic nature, abamectin is accumulating at high concentration in animal tissues fat and milk of lactating dams and then transfer to offspring [60, 65]. Previous pharmacokinetic studies have been completed on ivermectin (a dihydro derivative of avermectin). These studies reported accumulation and transfer of ivermectin from mothers to their offspring. It was found in milk, liver, blood, and the high residues found in liver tissue [66]. Milk has the high concentration (3-4 times) of ivermectin compared to blood [67].

Oral administration of spinosad daily for 28 consecutive days to male mice at doses equal to 350 mg/kg. b.wt induced significant decrease in body weight and increase in organ relative weight of kidney and spleen [68]. Breslin* et al*. [69] studied the effect of oral administration of spinosad at doses of 10, 50, and 200 mg/kg. b.wt. to pregnant rats and doses of 2.5, 10, and 50 mg/kg. b.wt. to pregnant New Zealand White rabbits during gestation period (21 day of rat and 28 days of rabbits). Spinosad at dose of 200 mg/kg. b.wt. caused lower body weight of rat on gestation day 12 (GD12) and decreased body weight gain on GD 7-10. Spinosad at dose of 50 mg/kg. b.wt. caused loss in body weight of rabbits at GD7-10 while the body weight gain decreased by 30% during the dosing period (GD7-20). Male and female rats are exposed to spinosad through feed in diet containing spinosad at concentration ranging from 0.003 to 0.4 % for 13 weeks. It induced reduction in body weight and mortality in rats fed in diet contains 0.4% spinosad [70]. Spinosad at doses of 0.02, 9, and 37.38 mg/kg. b.wt decrease body weight of treated rats [71].

Ibegbu* et al.* [72] found that body weight and relative kidney weight were significant increase in male rat exposed to aluminum phosphide at concentration 3 g of phostoxin tablet (1.5 hrs /day). Other studies showed the effect of phosphine on body weight of experimental animals at different concentrations. Newton* et al*. [55] studied the effect of phosphine gas on male and female rats. Rats were exposed to phosphine gas at levels 0.5, 1.5, and 4.5 mg/m^3^ for 6 hrs/day above 13 weeks. Inhalation exposure to phosphine gas caused significant decrease in body weight, especially at a high level of exposure. Other studies also reported reduction in body weight gains in rat exposed to phosphine [73, 74].

90-day subchronic study of azadirachtin at doses of 500, 1000, and 1500 mg/kg/day was completed in male and female rats [75]. There were no signs of toxicity, mortality, and changes in body and organ weights in male and female rats exposed to azadirachtin at all tested doses. Srivastava and Raizada [76] fed rats on diet contain azadirachtin at concentrations of 100, 500, and 1000 ppm (5, 25 and 50 mg/kg. b.wt.) for evaluation the toxicity during postnatal development for two generations. There was no toxicological effect of azadirachtin in body weight, liver, kidney, and brain of pups over two generations. It has been reported that* Bacillus thuringiensis (Bt)* does not induce any changes in body and organ weights in rat and rabbits [77, 78].

### 5.2. Hepatotoxicity

Liver is an important organ in the human body that plays an essential role in metabolism and detoxification of xenobiotic such as insecticides. Previous studies showed that the progress of liver toxicity and injury could be done through the two-stage course [79]. This two-stage contains direct interaction between toxin and hepatic cell, initiation of the injury [80], and development of the injury in a toxin-independent (Table 4). The mechanism of toxic liver injury in second stage is complete through (i) inflammatory of hepatic cells [81], (ii) increase of oxidative stress and lipid peroxidation [82], and (iii) cell membrane damage and leakage of degrading enzymes [80].

Pyrethrins or pyrethrum can be found as the dust of ground flowers or liquid as the crud extract. It can be used for control insects in agriculture and public health sectors. When pyrethrins exposed to sunlight, it breaks down quickly in the environment. The activities of hepatic microsomal enzymes, e.g., EPN detoxification (ethyl p-nitrophenyl thionobenzenephosphonate) such as p-nitroanisole demethylation and hexobarbital oxidation in addition to cytochrome P_450_, were increased in male rat administrated pyrethrum oral at dose of 200 mg/kg. b.wt. for 23 days [52]. The authors reported significant alteration hepatic pyrethrum metabolism and detoxification enzymes in the liver of male rats. It caused changes in liver enzymes and histopathological alterations in liver tissue in experimental animals such as mice, rats, rabbits, and dogs [53–56]. When rats were fed in feed, containing pyrethrins at 1000 mg/kg. b.wt. (high dose), liver dysfunction and damage were recorded [83]. Pyrethrins and other synthetic pyrethroids are neurotoxic compounds and can induce hepatotoxicity and liver damage in experimental animals. They caused alteration in liver biomarkers such as AST, ALT, ALP, LDH, albumin, and total protein in rat [10, 84, 85] and mice [86].

Abamectin (ABA) induced hepatotoxicity when administrated orally to male and female rats (*Rattus norvegicus*) at dose of 2.13 mg/animal/day for 28 days [87]. After the treatment period, the animal was kept 14 days without treatments as withdrawal period. ABA induced significant increase in plasma glucose and liver dysfunction biomarkers such as aspartase aminotransferase (AST), alanine transaminase (ALT), and *γ* glutamyl transpeptidase (*γ*-Gt) in male and female rats. It caused histopathological alteration in liver tissue in male and female rats. The authors reported that the residue of ABA (B1a) was found in plasma samples after 14 and 28 days of treatment while no residues was detected after 42 days. In contrast, B1a residue in liver was detected after 42 days. The presence of B1a residue in the liver for a longer period (42 days) compared with plasma (28 days) is due to the elimination of B1a from plasma by the fecal route and inhibition of g P-glycoprotein (P-gp) which lead to increased accumulation of ABA in the liver [88]. Abamectin (Vertemic 1.8 % EC) was administered to male rats at sublethal dose of 30 mg/kg. b.wt. (1/10 LD_50_) for 30 days (three doses per week) and at dose of 10 mg/kg. b.wt. (1/30 LD_50_) was administrated to another group for 210 days (one dose per week). ABA caused significant elevated liver enzymes in plasma (AST and ALT). It induced histopathological changes in liver tissue in a dose-dependent mater [63]. The toxic effect of ABA also was studied on female rats and their male and female pups during the lactation period [60]. ABA was administered orally to mothers at doses of 22.10, 11.05, and 2.21 mg/kg. b.wt from postnatal days 1-20 (PND1-20). It induced significant alteration in serum liver biomarkers such as AST, ALT, AL, and lactate dehydrogenase (LDH) in the mothers and their male and female pups in a dose-dependent manner. El-Gendy* et al*. [57] studied the acute toxicity of abamectin at sublethal dose (3.802 mg/kg. b.wt.) in male mice for 14 days. Significant increase in AST, ALT, and ALP of mice exposed to ABA was found along with histopathological alteration in liver tissue. Eissa and Zidan [78] evaluated the toxicity of abamectin on the liver of male albino rats. ABA caused significant alteration liver function biomarkers such as AST, ALT, alkaline phosphatase (ALP), acid phosphatase (ACP), albumin (ALB), and total protein (TP) in male rats administrated dietary doses of 1.81 and 0.181 mg/kg .b.wt. (1/10 and 1/100 LD_50_) for 30 days. The authors showed that the increase in liver enzymes could be due to the change of cell membrane permeability because of liver and hepatocyte damage. Other studies showed that the ABA could change the function of hepatic cells and induce liver damage and hepatotoxicity. It caused an increase in liver enzyme such as serum AST of rat exposed to the ABA at dose of 1 and 20 mg/kg. b.wt. [89].

Oral administration of spinosad daily for 14 consecutive days at doses equal to 35 and 350 mg/kg. b.wt caused liver damage in male mice. It induced significant elevation in liver function enzyme (AST and ALT) and in triglycerides [68]. Male and female rats are exposed to spinosad through feed in diet containing spinosad at concentration ranging from 0.003 to 0.4 % for 13 weeks. It induced hepatotoxicity and liver damage in rats' feed in diet containing 0.4% spinosad [70]. Zidan [90] studied the effect of exposure to spinosad on male rats. Rats were fed on wheat grains treated with spinosad at concentration 16 ppm for 90 consecutive days. At the end of the experimental period, biochemical parameters of the liver (AST, ALT, ALP, ACP, and albumin) were significant changes. Spinosad was evaluated for hepatotoxicity in male rats. Spinosad was orally administered for 8 weeks to male rats at doses of 0.02, 9, and 37.38 mg/kg. b.wt corresponding to Acceptable Daily Intake (ADI), No Observed Adverse Effect Level (NOAEL), and 1/100 LD_50_, respectively [91]. It caused significant alteration in liver function enzymes and histopathological alteration especially at high dose. In addition, it induced significant elevation in bilirubin (total and direct) in treated rats. Previous studies also reported hepatotoxicity of spinosad and alteration liver enzymes such as AST, ALT, and ALP on rats, rabbits, and Nile tilapia,* Oreochromis niloticus *[69, 92, 93].

Eissa and Zidan [78] evaluated the toxicity of* Bacillus thuringiensis* on the liver of male albino rats.* Bt* showed insignificant changes in liver dysfunction biomarkers of rat that has been administrated dietary doses of 300, 30 mg/kg. b.wt. (1/10 and 1/100 LD_50_). Meher* et al*. [77] reported that wettable powder formulation of* Bacillus thuringiensis* var. kenyae (B.t.k.) is nontoxic to rats given one single oral dose of 3, 4, and 5 ml Bt suspension (2.5x 10^7^ spores/ml) in acute toxicity study and toxicity was noted for 21 days. The authors found that acute oral LD_50_ in rats was higher than 5 ml of Bt containing (2.5x 10^7^ spores/ml) and LC_50_ in fish was higher than 1000 mg/L (2.5x 10^7^ spores/ml), respectively. In cases of rabbits, the acute dermal toxicity (LD_50_) was higher than 2.5 x 10^7^ spores/ml.* Bt* did not induce a significant alteration in the liver biomarkers, such as ALT, total protein, and glucose both in rats and in rabbits. In contrast, toxin of* Bacillus thuringiensis* (XenTari®) when administrated to pregnant rats at 370 mg/100 g equal to a dose of 20 mg/100 g of the protoxin (Cry subspecies Aizawai) altered liver function parameters and induced liver damage [94]. Moreover,* Bt* (Dipel®) can alter the defense behavior of hepatocytes and induce oxidative damage, lipid peroxidation, and damage of hepatocytes membrane in rats [95, 96].

Aluminum phosphide (ALP) is used as insecticides against many stored insects as fumigants. The inhalation toxicity of this compound is due to formation of phosphine gas because of contact of aluminum phosphide with moisture. ALP induced elevation in lactate dehydrogenase (LDH) in rats which received one single dose equal to 20 mg/kg. b.wt. It caused alteration in liver antioxidant enzymes and histological alteration [97].

### 5.3. Renal Toxicity

Although the liver is considered the major target organ for toxic compounds and plays an essential role of metabolism and detoxification, the kidney is similar, an essential site of damage after exposure to toxic compounds and their toxic metabolites. The kidney is playing an essential role with multifunction of elimination waste from blood, balance fluids in the body, and other important functions. Natural insecticides caused adverse toxic effects on the kidney in experimental animals (Table 5). Rats received pyrethrins at dose of 170 mg/kg. b.wt. for three moths showing degeneration in tubules and kidney damage [98].

Renal toxicity induced by abamectin was evaluated in male rats [78]. Rats were administered dietary ABA at doses of 1/10 or 1/100 LD_50_ for 30 repeated days. ABA induced significant changes in kidney biomarkers such as uric acid and creatinine concentration and histological alteration in kidney tissue. ABA induced significant increase in kidney markers such as urea and creatinine concentration and histological changes in male mice received dose equal to 1/10 LD_50_ for 14 days [83]. It caused an increase in kidney dysfunction biomarkers in male and female pups whose mothers are exposed to ABA at doses of 22.10, 11.05, and 2.21 mg/kg. b.wt during lactation period [60]. The authors reported also changes in kidney tissue structure, especially at high dose. ABA was orally administered to two groups of male rats at a dose equal to 0.44 mg /kg. b.wt. (1/20 LD_50_) and 0.87 mg/kg. b.wt. (1/10 LD_50_) for 4 weeks and 8 weeks, respectively. ABA induced significant increase in urea, uric acid, and creatinine concentrations in serum at 4 and 8 weeks in a dose-dependent manner. It caused histological changes in tissue structure and the effect increase with increasing exposure time [99].

Azadirachtin administered to male rat at doses of 500, 1000, and 1500 mg/kg. b.wt. for 90 days did not induce significant changes in kidney parameters or histological structure [75]. Srivastava and Raizada [76] evaluated the toxic effect of azadirachtin during postnatal development of rats. Rats were fed on diet containing azadirachtin at concentrations 100, 500, and 1000 ppm (5, 25, and 50 mg/kg. b.wt). No adverse effects were recorded in rats over two generations with no effect on kidney function or tissue. The adverse toxic effect of spinosad at dose 35 and 350 mg/kg on some biochemical and histological parameters of kidney of male mice was evaluated. Significant increase in urea concentration in serum of male mice received 350 mg/kg spinosad. Histopathological investigation revealed cytoplasmic degeneration and cell necrosis in kidney [68]. Spinosad in two studies conducted on male and female mice fed in feed containing 0.005, 0.015, 0.045, and 0.12% and 0.0025, 0.008, or 0.036% spinosad, respectively, for 13-week and 18-month chronic studies. The histopathological alteration in mouse kidney was noted at doses more than 0.015% [92]. Spinosad was administrated to male rat at doses equal to ADI, NOAEL, and 1/100 LD_50_ (0.02, 9.0, and 37.38 mg/kg. b.wt.) for 8 weeks via the oral route. Spinosad at high two doses (9 and 37.38 mg/kg. b.wt.) induced significant changes in uric acid concentration of male rats and histopathological alteration such as degeneration, necrosis [91].

Lemos* et al*. [94] reported that* Bacillus thuringiensis* toxin (XenTari®) induces necrosis, degeneration in tubules and proliferative glomerulonephritis in kidney of pregnant rats received 370 mg/100 g equal to a dose of 20 mg/100 g of the protoxin (Cry subspecies Aizawai). The authors explained the alteration in the kidney after exposure to* Bt* toxins is due to the effect of toxins on the immune system through the proliferation of mesangial cells and their infiltration in the kidney tissue. The Cry1Ia12 entomotoxin from a Brazilian* Bt.* strain was used to evaluate the adverse effects of diet Cry1Ia12 protein for 10 days to rats on blood urea nitrogen and kidney [100]. The authors found no significant changes in blood urea nitrogen and no histopathological alteration in kidney tissue of the rats diet with 0.1% of the Cry1Ia12 toxin for 10 days.

Morgan* et al*. [101] evaluated the inhalation toxicity of phosphine (PH3) on males and females rats and mice. Male and female mice and rats were exposed to concentration 1,5,10 ppm and males and females were exposed to 1.25, 2.5, and 5 ppm for 4 days and 14 days, respectively. Phosphine at high concentration (10 ppm) caused an increase in concentration of urine nitrogen in males and females rats and mice. It caused histopathological alteration in kidney such as degeneration and necrosis of renal tubule epithelium. The authors explained that the increase in urine nitrogen could be due to the toxic effect of phosphine in kidney that decreased renal blood flow and may be due to the kidney dehydration.

It has been confirmed that* Lecanicillium muscarium *strain Ve6 is not a plant and human pathogens. As shown in the literature,* Lecanicillium muscarium (Lecanicillium *spp) was previously widely known as* Verticillium lecanii* (*Verticillium *spp.). To the best of our knowledge, there are no signs of toxicity or mortality and no fungi were detected in organs of rats and mice treated with the level ranged from 6.9 x 10^6^ to 3.0 x 10^8^ spores/animal [102].

### 5.4. Hematotoxicity

The toxic effect of insecticides on hematological parameters is extensively used to study the effect on blood components. Insecticide can induce hematotoxicity in experimental animals and changes in hemoglobin (Hb) concentration, red blood cells count (RBCs), white blood cells count (WBCs), mean corpuscular volume (MCV), and mean corpuscular hemoglobin (MCH) and other components. Abamectin was administered to male rats at dose equal to 1/10 and 1/100 LD_50_ daily for 30 days. Some hematological parameters in blood were decreased such as Hb, RBCs and WBCs [78]. The acute toxicity of ABA was investigated in male mice [57].

Mice was exposed to ABA at dose equal to 1/10 LD_50_ for 14 days. ABA induced significant changes in hematological parameters in the blood of treated mice. It caused significant reduction in Hb concentration, packed cell volume (PCV) and RBCs and increase in WBCs, MCV, and MCH. Mansour* et al*. [91] evaluated the hematotoxicity of spinosad as single and repeated dose for 21 days on male rats. Rats were administrated spinosad at doses of 0.02 (ADI), 9 (NOAEL), and 37.38 mg/kg. b.wt. (1/100LD_50_). Spinosad induced decrease in RBCs and Hb while it induced increase in WBCs, lymphocyte concentration, and granulocyte concentration. Other studies reported hematotoxicity of spinosad to experimental animals; for example, it increases WBCs in female rats after 18 months of exposure [70]. It increases WBCs in female rats after receiving 0.036 % and 0.024 for 12 months [92].

Biosafety studies have confirmed that exposures to* Bt* spore-crystals induce minor toxic effects to mammals. It has been reported that product of Bt is noninfectious and they are toxic to mammals at doses above 10^8^ and 10^11^ colony forming units (CFU) per mouse and per human, respectively [103]. Male and female mice were orally exposed 72h to* Bt* Cry1Ia and Cry1Ba6.* Bt* caused significant decrease in MCH whereas insignificant changes were noted in RBCs, Hb, and HCT [104]. Significant reduction was found in MCV after treatment by Cry10Aa (5 × 10^9^ spores/kg), Cry10Aa (1×10^10^ spores/kg), and Cry1Ba6. Other studies reported hematotoxicity of natural insecticides such as abamectin, spinosad, azadirachtin, pyrethrins, and phosphine on experimental animals [78, 101, 105–107].

### 5.5. Reproductive Toxicity

Several researchers reported reproductive toxicity of natural insecticides on experimental animals. Abamectin was administered to male rats orally at dose of 10 mg/kg. b.wt. for six weeks [108]. It induced alteration in testis function and histological alterations. The results of reproductive toxicity induced by pyrethrins are different depending on animal species. Normal litters were obtained from pregnant rabbits who fed on diet containing pyrethrin up to 90 mg/kg during the superficial period of pregnancy. No mortality was recorded in rabbit litters at birth after exposing to pyrethrins. When rats fed on a diet containing pyrethrins at 5000 mg/kg for 21 days before their initial mating, reduction in the weight of birth litter was recorded [109]. Normal litters also were recorded in rabbits fed at moderate dose of pyrethrins around 90 mg/kg. b.wt. during pregnancy, while reduction in pup weights at birth was noted in mothers fed to pyrethrins at dose of 500 mg/kg. b.wt. (high doses) for 21 days before their first mating [83]. In rabbits exposed to pyrethrins, there are no birth defects [110].

ABA caused an increase in the concentration of testosterone in plasma of male rats and decrease in sperm count and sperm motility [111]. ABA induced reduction in springs of male rats after expose for 30 days (subacute) and 210 days (subchronic) [63]. Histological alteration in the testes of male rats was recorded after exposure to ABA at doses of 1.19, 1.87, and 2.13 mg/animal/day for 180 days. It caused infiltration, congestion in the blood vessels, and hemorrhage [112]. Oral administration of male rats for one week by 1 mg/kg/day (low dose) and 4 mg/kg/day (high dose) for 6 weeks induced changes in spermatogenesis [113, 114]. It has been reported that ABA can induce histopathological alteration in the testes of male rats, such as degeneration, edema, and necrosis of spermatogonia cells lining seminiferous tubules associated and decreased number of spermatogenic elements [63, 112].

Srivastava and Raizada [76] evaluated the reproductive toxicity of azadirachtin on rats throughout postnatal development of rats for two generations. Rats were fed on diet containing azadirachtin at concentrations 100, 500, and 1000 ppm that is corresponding to 5, 25, and 50 mg/kg. b.wt. Results showed no toxic effect of azadirachtin on reproductive function on rats and over two generations. Technical azadirachtin has not produced any adverse effects on reproductive function and data were comparable to control animals over two generations. Administration ABA to rats at dose of 0.4 mg/kg. wt. caused increase in stillbirths and decrease pups viability, lactation, and pups weights. This finding suggests that the ABA could have the possibility of inducing reproductive effects at high enough doses [115]. At dose toxic to mice and rabbit mothers, ABA can cause cleft palate in the offspring [116]. In rat-administered ABA at 1 mg/kg/ day, no birth defects were recorded and the teratogenic effect caused at high toxic doses to mother [115].

Hanley* et al*. [117] evaluated the reproductive effect of spinosad on rats for two continual generations. Rats were administrated spinosad at doses of 3, 10, and 100 mg/kg. b.wt. via diet dally for 2 generations. Treatment with spinosad in diet at high dose (100 mg/kg. b.wt.) causes maternal toxicity and adverse effect on the offspring. The authors reported no significant effects of spinosad at lower doses. Breslin* et al*. [69] found the same trend in another study on pregnant rats and rabbits administrated by gavage spinosad at doses of 10, 50, and 200 mg/kg. b.wt. for rats and 2.5, 10, and 50 mg/kg. b.wt. for rabbits, respectively. The authors reported no maternal effect of spinosad at lower doses. Spinosad not induced signs of developmental toxicity at all tested doses. The NOEL (No Observed Effect Levels) of maternal was 50 mg/kg. b.wt. of rats and 10 mg/kg. b.wt of rabbits.

It has been reported that* Bt* do not have any reproductive effects or birth defects in mammals and also no available studies regarding this issue [118]. There are also no available data and studies on the reproductive and developmental toxicity of phosphine [119].

### 5.6. Oxidative Stress

Oxidative stress is one of the common explanations for the mechanism of toxic action of pesticides, especially when exposed to low doses and for a long time. It is caused because of a disturbance in the balance between antioxidant defenses in the body and the level of free radicals or reactive oxygen species (ROS). Oxidative stress occurs when the level of ROS increases compared to the antioxidant defense mechanisms. When ROS is increased, the damage is occurring to lipid “lipid peroxidation”, protein, and genetic materials such as DNA. Moreover, it has played an important role in the damage of cell membrane, cell, tissue, and organ (Table 6). The effect of exposure to spinosad on oxidative stress biomarkers in male rats was evaluated [120].

Rats were given spinosad via oral route at dose of 347.49 mg/kg. b.wt. (1/20 LD_50_) for four weeks. It caused inhibition in the activity of glutathione-S-transferase (GST), catalase (CAT), and superoxide dismutase (SOD) and level of glutathione (GSH). It caused a decrease in the activity of glutathione peroxidase (GPx) and level of reduced and lipid peroxidation in the liver. The authors suggested that the hepatotoxicity of spinosad could be due to the formation of ROS in hepatic cells. It induced oxidative stress in the liver of Nile tilapia (*Oreochromis niloticus*) after exposure to 25, 50, and 75 mg/L for 24, 48, and 72 h [93]. It causes significant alterations on oxidative stress markers in the liver of Nile tilapia.

Abamectin caused alteration in oxidative stress markers in male and female pups whose mothers are exposed to 22.10, 11.05, and 2.21 mg/kg. b.wt. during lactation period [60]. The authors reported also changes in kidney tissue structure, especially at high dose. ABA was orally administered to rats at dose of 30 mg/kg. b.wt for 30 days [121]. It caused reduction in the activity of SOD, CAT, GST, and GSH and increase in the level of these biomarkers in kidney and brain of treated rats. Other studies also reported oxidative stress induced by abamectin in testis of male rats [113] and in the spleen of pigeon [122]. The effect of ABA on brain of rats was studied by Abdel-Razik and Hamed [64]. Rats were orally administered ABA at dose of 3.3 mg/kg. b.wt. as single dose and oxidative stress (SOD, GSH, and LPO) was determined after 4, 24, and 36 h of treatment. ABA induced oxidative stress that caused a significant reduction in SOD activity and level of GSH. It caused also significant increase in the level of LPO. Hmani* et al*. [123] evaluated the oral toxicity of* Bt* toxin (Vip3Aa16) on oxidative stress in liver and kidney of mice. Mice were administrated* Bt* toxin at dose of 2500, 5000, and 7500 mg/kg. b.wt. for 14 days. There were no significant changes in oxidative stress parameters such as SOD, H_2_O_2_ (hydrogen peroxide) and LPO in the liver and kidney of exposed mice.

Phosphine (produced from aluminum, magnesium, and zinc phosphide) was evaluated for toxic effects on male rats [124]. Rats were administered intraperitoneally (i.p.) with phosphine at dose of 4 mg/kg. b.wt. as single dose; the kidney and heart were taken after 30 min of exposure. Phosphine induced significant reduction in the level of GSH and activity of SOD, CAT, and GPx. It caused a significant increase in LPO in both tissues. In another study by the same authors [125], rats were treated i.p. with phosphine at dose of 2 mg/kg. b.wt. and oxidative stress parameters were evaluated in brain, liver, and lung after 3 mint of treatment. PH_3_ induced significant reduction in GSH and the increase in LPO concentration.

### 5.7. Neurotoxicity

The toxic effect of some insecticides in the nervous system was reported both in insects and in mammals. Most insecticides with neurotoxicity are not highly selective and cause neurotoxic effect to nontarget organisms such as beneficial insects, animals and humans. It has been reported that insecticides cause neurotoxicity and kill insects by an effect on their nervous system and can also induce neurotoxic effect in animals and humans. The neurotoxic effect of most insecticides can be induced their acute toxicity at high dose of exposure or can cause chronic neurodegenerative diseases such as Parkinson's disease [126]. The mechanism of neurotoxicity of some natural insecticides was reported. For example, pyrethrins are neurotoxicant [23], affect sodium channels [24], and affect gamma amino butyric acid (GABA) [26]. Spinosad is a neurotoxin and the effect on the nicotinic acetylcholine receptor and gamma amino butyric acid (GABA) receptor [16] in the site different from imidacloprid and nicotine. The ABA has an effect on the nervous system by blocking the ion tropic *α*-amino butyric acid (GABA) [35]. Phosphine also has neurotoxicity and causes nerve poison [27].

It has been reported that pyrethrins are poisonous insecticides to animals and humans [127]. It caused neurotoxic action in the nerves system [23] and effect on animals activities. For example, rats administered pyrethrins showed some signs of toxicity, e.g., difficulty or quick inhalation, incoordination, unbalanced, tremors, aggression, and sensitivity to external stimuli [128].

Male and female rats were administered abamectin at dose of 0.5, 1.5, and 6 mg/kg. b.wt. as single oral dose and observed for 14 days [129]. ABA caused signs of neurotoxicity that persisted in some female rats after two or three days of treatment and reduced motor activity. It caused decrease in brain weight in female rats but without any histopathological changes [130]. Other studies reported neurotoxic effects of ABA in experimental animals, especially at high doses. The developmental neurotoxicity by ABA (emamectin benzoate) was recorded in the F_1_ offspring whose mother exposed to ABA during gestation and lactation periods. The authors reported that the NOAEL is 0.6 mg/kg/day for developmental neurotoxicity of ABA [131].

Marrs [132] reported that spinosad has not specific neurotoxicity in experimental animals such as rats in acute and chronic studies. The author also reported that the toxic effect of spinosad is closely similar to toxic effect of spinosad A. Spinosad did not induce neurotoxicity in acute, subacute, and chronic toxicity studies in rats. According to the US EPA [133], spinosad did not cause neurotoxicity in rats in acute, subchronic, or chronic toxicity studies.

Previous studies showed that phosphine could inhibit acetylcholine esterase (AChE), which lead to increase the neurotransmitter acetylcholine [134, 135]. Phosphine like other OPIs caused inhibition in AChE and neurotoxicity in mammals. In inhalation exposure, phosphine induced neurotoxicity in rats after acute and subchronic (90 days) exposure. The NOAEL was 38 ppm in the acute neurotoxicity study [136].

### 5.8. Mutagenicity and Genotoxicity

Mutagenicity in general means that all chemicals or physical agents are able to induce changes in the genetic material, usually deoxyribonucleic acid DNA in organism, e.g., bacteria, animals, humans, and others. This change leads to increases in the rate of mutations over the natural level. In contrast, genotoxicity means the ability of chemical agents to induce damage in the genetic materials that lead to loss of genetic information in cell and inducing mutations that could lead to cancer. In this regard, not all genotoxic agents are mutagens but all mutagens are genotoxic agents. ABA does not seem to be mutagenic. It has a negative mutagenic effect in mice and rats. Moreover, it is shown to be nonmutagenic in the Ames test [137]. Indifference, abamectin has potential genotoxic effects on the Chinese hamster ovary (CHOK1) cells [138]. Spinosad has been reported no mutagenic effect by EPA [139] while Mansour* et al*. [71] found that spinosad has genotoxic effect in rat bone marrow cells. The authors explain the genotoxic effect of spinosad is due to the active ingredient and or the inert materials used in insecticide formulation. None of the genotoxicity studies showed mutagenic activity associated with spinosad [132]. de Souza Freire* et al*. [104] evaluated three Cry-endotoxins (BtCry1Ia, BtCry10Aa, and BtCry1Ba6) from* Bt *(Bt) on mice to study their effect on genetic material. The three tested endotoxins did not induce any changes in micronucleus and polychromatic erythrocytes in mice. Phosphine gas is not clastogenic and does not induce mutations in mice after exposure to 14 days [140]. However, phosphine does not cause cytogenetic effects in experimental animals such as male mice and rats after subacute exposure (11-12 days) [141]. Limited data about the genotoxic effect of natural pyrethrins was found. Negative results were recorded of natural pyrethrins by using the Ames test on* Salmonella* and* Escherichia coli* [142]. In addition, it has been reported that pyrethrin did not induce gene mutation in mice and at high concentration and the frequency of mutation was significant increase [143]. In contrast, several studies reported mutagenic and genotoxic effect of synthetic pyrethroids on experimental animals [84, 144, 145].

Neem extract (most active compound is azadirachtin) was evaluated for cytogenetic in murine germ cells [146]. The extract caused an increase in chromosomal aberration of spermatocytes and increase up normal sperms. Therefore, azadirachtin can induce mutagenicity and genotoxicity. In another study on male mice, crude leaf extract of neem (*Azadirachta indica*) at dose of 0.5, 1.0, and 2.0 g/kg. b.wt. for one week induced chromosomal aberration in bone marrow [147].

### 5.9. Carcinogenicity

A carcinogen means that any material can promote carcinogenesis (formation of cancer). This effect can be due to the ability of this substance to induce damage in the genome and or to the disturbance of cellular metabolic processes. Pyrethrins were studied for carcinogenicity on experimental animals. In one of these studies, rats were exposed to pyrethrins (plant extract) at doses of 100, 1000, and 3000 mg/kg. b.wt. (moderate and very high doses) through feeding for 104 weeks. Pyrethrins increased the noncancerous (benign) thyroid tumors in females at all doses and in males at high to very high doses [148]. The same authors reported that pyrethrins induced development in ovarian and benign liver tumors in female rats fed on high doses (3000 mg/kg. b.wt.). This dose developed benign parathyroid tumors and benign skin lesions in male rats [148]. In a different study, pyrethrins at dose less than 10 mg/kg. b.wt. showed no increase in tumor in rats [128]. Therefore, US EPA classified pyrethrins as “likely to be carcinogenic to humans by the oral route” [148].

It has been reported that abamectin is not carcinogenic in mice and rats [116]. Also, according to US EPA [51], ABA is noncarcinogenic to human and classified in-group E. Spinosad was also classified as noncarcinogenic to mice and rats [132]. In another study spinosad was not carcinogenic to male and female rats at dose near to 0.05% [70]. There is no available information regarding the carcinogenic effect of phosphine in human. Moreover, rats exposed to phosphine via diets showed no carcinogenic effect [140, 148]. Therefore, EPA [140] has not classified phosphine as human carcinogenicity (Group D).* Bt* does not have any chronic toxicity and carcinogenic effects [118]. There is also no indication that* Bt* causes reproductive effects or birth defects in mammals.

## 6. Natural Insecticides and Organic Farmers

From this review and previous studies, it can be reported that some natural insecticides have adverse toxic effects in experimental animals. It can induce alterations in biochemical, hormonal, reproductive, and oxidative stress biomarkers. It causes also cytotoxic, autogenetic, genotoxic, and carcinogenic effects in experimental animals [60, 71, 78, 118, 148]. Therefore, some natural compounds not safe and the term “natural” not mean compounds are safe.

## 7. Conclusion

The natural insecticide content is botanical insecticides (e.g., pyrethrins and azadirachtin), microbial insecticides (e.g.,* Bacillus thuringiensis, Lecanicillium muscarium*, abamectin, and spinosad), mineral oils, and minerals. The selectivity and safety of natural insecticides are not absolute and some natural compounds are toxic and induced adverse effects to experimental animals. It can induce hepatotoxicity, renal toxicity, hematotoxicity, reproductive toxicity, neurotoxicity, and oxidative stress. It can induce mutagenicity, genotoxicity, and carcinogenicity in mammals. Some natural insecticides and active compounds from essential oils are classified in categories Ib (Highly hazardous) to U (unlikely toxic). Therefore, all natural insecticides are not safe and the term “natural” does not mean that compounds are safe. In this respect, the term “natural” is not synonymous with “organic” and not all-natural insecticide products are acceptable in organic farmers.

## Figures and Tables

**Table 1:** Acute toxicity hazard categories of pesticides and distribution of pesticides registered in Egypt in each categories [20].

Category or class	Acute LD_50_ mg/kg body weight of the rat		Classification by hazardous
	Oral	Dermal	
Ia	< 5	< 50	Extremely
Ib	5–50	50–200	Highly
II	50–2000	200–2000	Moderately
III	Over 2000	Over 2000	Slightly
U	5000 or higher		Unlikely

**Table 2:** Toxicity of some essential oil compounds to experimental animals.

Compound	Animal	Route	LD_50_ mg/kg. b.wt.	WHO category
Category II (Moderately hazardous), 50-2000 mg/kg				

Thujone	Mice	Subcutaneous	87.5	II
Pulegone	Mice	Intraperitoneal	150	II
3-Isothujone	Mice	Subcutaneous	442.2	II
Apiol	Dogs	Intravenous	500	II
2-Acetonaphthone	Mice	Oral	599	II
2-Methoxyphenol	Rats	Oral	725	II
Thymol	Rats	Oral	980	II
Linalool	Rats	Oral	>1000	II
Cinnamaldehyde	Guinea pigs	Oral	1160	II
Methyl eugenol	Rats	Oral	1179	II
Dillapiol	Rats	Oral	1000-1500	II
Anisaldehyde	Rats	Oral	1510	II
(+) Carvone	Rats	Oral	1640	II
*γ*-terpinene	Rats	Oral	1680	II
Thymol	Mice	Oral	1800	II
Methyl chavicol	Rats	Oral	1820	II

Category III (Slightly hazardous), over 2000 mg/kg				

trans-Anethole	Rats	Oral	2090	III
Cinnamaldehyde	Rats	Oral	2220	III
Maltol	Rats	Oral	2330	III
1,8-Cineole	Rats	Oral	2480	III
Eugenol	Rats	Oral	2680	III
Menthol	Rats	Oral	3180	III
Terpinen-4-ol	Rats	Oral	4300	III
d-Limonene	Rats	Oral	4600	III
Citral	Rats	Oral	4960	III

Category U (unlikely to present acute hazard), 5000 mg/kg				

Myrcene	Rats	Oral	5000	U

**Table 3:** Effect of exposure to some natural insecticides on body and organs weight in experimental animals.

Insecticide	Treatment	Dose mg/kg. b.wt. or animal/day	Duration (days)	Test animal	Organ	Reference
Pyrethrum	oral	200	23	rat (♂)	body*ꜛ*, liver*ꜛ*,	[52]
Abamectin	oral	3.802	14	mice (♂)	body*ꜜ*, liver*ꜛ*, kidney*ꜛ*	[57]
Abamectin	oral	22.10	21	rat (♀, ♂)	body*ꜜ*, liver*ꜛ*, kidney*ꜛ*	[60]
Spinosad	oral	350	28	mice (♂)	body*ꜜ*, kidney*ꜛ*, spleen,*ꜛ*	[68]
Spinosad	oral	10, 50, 200	21	rat (♀, ♂)	body*ꜛ*	[69]
		2.5, 10, 50	28	rabbits (♀)	body*ꜛ*	
*Bt *	fed	300, 30	30	rat (♂)	body, liver, kidney	[78]
Phosphine	inhalation	3 g of phostoxin tablet (1.5 hrs/day)	7	rat (♂)	body*ꜛ*, kidney*ꜛ*	[72]
Phosphine	inhalation	0.5, 1.5 and 4.5 mg/m3 (6hrs/day)	13 (weeks)	rat (♀, ♂)	body*ꜜ*	[55]
Azadirachtin	oral	500, 1000, 1500	90	rat (♀, ♂)	body, liver, kidney, testis, spleen, heart, lung	[75]
*Bt *	oral, dermal	3,4,5 ml	1	rat (♀,♂), rabbit (♀,♂)	body, liver, kidney	[77]
Azadirachtin	fed	5, 25, 50	70	rat (♀, ♂)	body, liver, kidney, brain	[76]

Male** (**♂), female (♀), increase “*ꜛ*”, decrease “*ꜜ*”, and no change “without symbol”. *Bt*: *Bacillus thuringiensis.*

**Table 4:** Effect of exposure to some natural insecticides on liver function biomarkers in experimental animals.

Insecticide	Treatment	Dose mg/kg. b.wt. or animal/day	Duration (days)	Test animal	Sample	Liver function biomarkers	Reference
Abamectin	Oral	2.13	28	rat (♀, ♂)	plasma, liver	AST*ꜛ*, ALT*ꜛ*, ALP*ꜛ*, glucose*ꜛ*	[87]
Abamectin	Fed	1.81, 0.181	30	rat (♂)	serum	AST*ꜛ*, ALT*ꜛ*, ACP*ꜛ*, ALP*ꜛ*, ALB*ꜜ*, TP*ꜜ*	[78]
*Bt*	Fed	300, 30	30			AST, ALT, ACP, ALP, ALB, TP	
Abamectin	Oral	30, 10	30, 210	rat (♂)	plasma	AST*ꜛ*, ALP*ꜛ*, ALB*ꜛ*, TP*ꜛ*	[63]
Abamectin	Oral	3.802	14	mice (♂)	serum	AST*ꜛ*, ALT*ꜛ*, ALP*ꜛ*	[57]
Spinosad	Oral	35, 350	28	mice (♂)	serum	ALT*ꜛ*, AST*ꜛ*, ALB*ꜛ*, TP, TG	[68]
Pyrethrum	Oral	200	23	rat (♂)	liver	EPN, Cytochromes P_450_	[52]
Spinosad	Fed	16 ppm	90	rat (♂)	serum	AST*ꜛ*, ALP*ꜛ*, ALT*ꜛ*, ACP*ꜛ*	[90]
Spinosad	Oral	0.02, 9, 37.38	56	rat (♂)	serum	AST*ꜛ*, ALT*ꜛ*, ALP*ꜛ*	[91]
Bt	Oral	3,4,5 ml	1	rat (♀, ♂)	serum	ALT, TP, glucose	[77]
ALP^*∗*^	Oral	20	1	rat (♂)	liver	LDH*ꜛ*	[97]

Male** (**♂), female (♀), increase “*ꜛ*”, decrease “*ꜜ*”, and no change “without symbol”. ALP: alkaline phosphatase; ACP: acid phosphatase; ALB: albumin; and TP: total protein. TG: total triglycerides; EPN detoxification: ethyl p-nitrophenyl thionobenzenephosphonate such as p-nitroanisole demethylation and hexobarbital oxidation. *Bt*: *Bacillus thuringiensis*; ALP^*∗*^: aluminum phosphide; LDH: lactate dehydrogenase.

**Table 5:** Effect of exposure to some natural insecticides on kidney function biomarkers in experimental animals.

Insecticide	Treatment	Dose mg/kg. b.wt. or animal/day	Duration (days)	Test animal	Sample	kidney function biomarkers	Reference
Pyrethrum	Oral	170	90	rat (♀, ♂)	kidney	-* *-* *-	[98]
Abamectin	Fed	1.81, 0.181	30	rat (♂)	serum	uric acid*ꜛ*, creatinine*ꜛ*	[78]
Abamectin	Oral	3.802	14	mice (♂)	serum	urea*ꜛ*, creatinine*ꜛ*	[57]
Abamectin	Oral	2.21, 11.05, 22.10	21	rat (♀, ♂)	serum	uric acid*ꜛ*, creatinine*ꜛ*	[60]
Azadirachtin	Oral	500, 1000, 1500	90	rat (♂)	serum	uric acid, creatinine	[75]
Spinosad	Oral	35, 350	28	mice (♂)	serum	urea	[68]
Azadirachtin	Fed	5, 25, 50		rat (♀, ♂)	serum	urea, creatinine	[76]
Spinosad	Oral	0.02,9,37.38	8 weeks	rat (♂)	serum	uric acid*ꜜ*	[91]
Bt (toxin)	Oral	20 mg/100g	21	rat (♀, ♂)	kidney	kidney	[94]
Bt ( Cry1Ia12 toxin)	Diet	0.1%	10	rat (♂)	kidney	urea nitrogen	[100]
Phosphine	inhalation	1,5,10 ppm; 1.25, 2.5, 5 ppm	4 14	rat (♀, ♂);mice (♀, ♂)	serum	urine nitrogen*ꜛ*	[101]
Abamectin	Oral	0.44, 0.87	4,8 weeks	rat (♂)	serum	urea*ꜛ*, uric acid*ꜛ*, creatinine*ꜛ*	[99]

Male** (**♂), female (♀), increase “*ꜛ*”, decrease “*ꜜ*”, no change “without symbol”, and *Bacillus thuringiensis* (*Bt*).

**Table 6:** Oxidative stress induced by some natural insecticides in experimental animals.

Insecticide	Treatment	Dose mg/kg. b.wt. or animal/day	Duration (days)	Test animal	Sample	Oxidative stress biomarkers	Reference
Spinosad	Oral	347.49	28	rat (♂)	liver	SOD*ꜜ*, CAT*ꜜ*, GST*ꜜ*, GPx*ꜛ*, GSH*ꜜ*, LPO*ꜛ*	[120]
Spinosad	dipping	25, 50, 75 mg/L	24, 48, 72 h.	fish	liver	GST*ꜛ*, GPx*ꜛ*, GSH*ꜜ*, LPO*ꜛ*	[93]
Abamectin	Oral	2.21, 11.05, 22.10	21	rat (♀, ♂)	liver, kidney	SOD*ꜛ*, GST*ꜛ*, LPO*ꜜ*	[60]
Abamectin	Oral	30	30	rat (♂)	kidney, brain	SOD*ꜜ*, CAT*ꜜ*, GST*ꜜ*, GSH*ꜜ*, LPO*ꜛ*	[121]
Abamectin	Oral	1,4	7,28	rat (♂)	testis	4-HNE*ꜜ*, PAR*ꜜ*, *ꜜ* PARP*ꜜ*	[113]
Abamectin	Fed	20,40,60	30,60,90	pigeon	spleen	SOD*ꜜ*, GPx*ꜜ*, T-AOC*ꜜ*, LPO*ꜛ*	[122]
*Bt* toxin “Vip3Aa16”	Oral	2500, 5000, 7500	14	mice	liver, kidney	SOD, LPO, H_2_O_2_	[123]
Abamectin	Oral	3.3	4, 24, 48 h.	rat	brain	SOD*ꜜ*, GSH*ꜜ*, LPO*ꜛ*	[64]
Phosphine	i.p.	4	30 min	rat	kidney, heart	SOD*ꜜ*, CAT*ꜜ*, GPx*ꜜ*,,GSH*ꜜ*, LPO*ꜛ*	[124]
Phosphine	i.p.	2	30 min	rat	brain, liver, lung	GSH*ꜜ*, LPO*ꜛ*	[125]

Male** (**♂), female (♀), increase “*ꜛ*”, decrease “*ꜜ*”, and no change “without symbol”. Bt*: Bacillus thuringiensis*; GST: glutathione-S-transferase; CAT: catalase; SOD: superoxide dismutase; LPO: lipid peroxidation; GSH: glutathione; T-AOC: total antioxidant capability. 4-HNE: 4-hydroxy-2-nonenal, PAR: poly(ADP-ribose), PARP: poly(ADP-ribose) polymerase; i.p.: intraperitoneally.
